# Successful Acute-Phase Rehabilitation Leading to Activities of Daily Living Recovery in a Patient With Hypoxic Encephalopathy Following Prolonged Cardiopulmonary Arrest: A Case Report

**DOI:** 10.7759/cureus.79910

**Published:** 2025-03-02

**Authors:** Yuki Watanabe, Risa Suzuki

**Affiliations:** 1 Department of Rehabilitation Medicine, Shizuoka General Hospital, Shizuoka, JPN; 2 Faculty of Human Sciences, Waseda University, Tokorozawa, JPN

**Keywords:** adl (activities of daily living), cardiopulmonary arrest, high-frequency interventions, orthotic-therapy, physiotherapy, short-term

## Abstract

Hypoxic encephalopathy due to prolonged cardiopulmonary arrest is associated with poor neurological prognosis, often leading to significant impairment in activities of daily living (ADL) and social reintegration. Rehabilitation for patients experiencing cardiac arrest lasting over 15 minutes is rarely successful. This case report describes a 29-year-old male patient with hypoxic encephalopathy following a 19-minute cardiopulmonary arrest while in a sauna, who achieved substantial ADL recovery through intensive acute-phase rehabilitation. Cardiopulmonary resuscitation (CPR) was initiated 13 minutes after collapse, and ventricular fibrillation was identified six minutes later by an automated external defibrillator (AED) and successfully treated with a single defibrillation. On arrival at the hospital, laboratory findings revealed metabolic acidosis and myocardial dysfunction, with an ejection fraction of 35%. The patient had a history of Wolff-Parkinson-White (WPW) syndrome. Brain MRI with diffusion-weighted imaging (DWI) showed high signal intensity in the occipital lobe and medial thalamus, leading to a diagnosis of hypoxic encephalopathy. The patient's initial neurological assessment indicated severe impairment, with a Glasgow Coma Scale (GCS) score of E4/VT/M6, a modified Rankin Scale (mRS) score of 5, and a Cerebral Performance Category (CPC) score of 4. He experienced generalized seizures requiring continuous anticonvulsant therapy. Rehabilitation was initiated on hospital day (HD) 3 but was discontinued on HD 15 due to poor consciousness. It was resumed on HD 27 after neurological improvement. The rehabilitation strategy focused on short-interval, high-frequency interventions, beginning with postural control exercises and passive range of motion therapy. As the patient's muscle strength improved, wheelchair mobility training and standing exercises were introduced. Gait training commenced with a body-weight support walker on HD 67, transitioning to long-leg orthotic therapy and progressive gait exercises. By HD 123, the patient's lower limb muscle strength improved from Manual Muscle Testing (MMT) grade 1 to 4, and the Barthel Index (BI) score increased from 0 to 45. He was able to walk 50 meters with light assistance and perform ADLs with minimal support. The patient was subsequently transferred to a rehabilitation hospital for further recovery. His significant recovery was attributed to early and intensive rehabilitation, which prevented disuse syndrome and facilitated neuroplasticity. Short-duration, high-frequency interventions played a crucial role in improving cardiovascular endurance, maintaining musculoskeletal function, and supporting cognitive recovery. The use of assistive devices, particularly long-leg orthoses, was instrumental in facilitating ambulation by compensating for lower limb weakness. Additionally, interdisciplinary collaboration, including occupational therapy and nursing interventions, played a critical role in optimizing functional outcomes. Despite prolonged cardiopulmonary arrest and an initially severe neurological prognosis, the patient demonstrated remarkable functional recovery, challenging conventional expectations. Thus, structured and intensive physiotherapy may significantly improve functional outcomes, even in patients with initially poor prognostic indicators.

## Introduction

Sudden cardiac arrest is a significant cause of death, and in Japan, 120,000 people suffer from out-of-hospital cardiac arrest each year. The rate of resuscitation for out-of-hospital cardiac arrest is low [1]. Even for patients who resume their heartbeats through resuscitation, the rate of social reintegration due to cardiac arrest syndromes, such as heart failure, infection, and hypoxic encephalopathy, is low [2]. Hypoxic encephalopathy is a general term for brain dysfunction caused by a temporary interruption in the supply of oxygen and glucose to the central nervous system [3]. Furthermore, it is associated with the duration of cardiopulmonary arrest and is considered to have a poor functional prognosis over time. In addition, even if resuscitation is performed, severe disturbance of consciousness is observed from an early stage, and even if consciousness improves, cognitive dysfunction and severe motor dysfunction remain [4].

There are many case reports on rehabilitation after cardiac arrest, but there are few reports on poor neuroprognosis after cardiac arrest of more than 15 minutes is reintegrated into society [5]. In cases of out-of-hospital cardiac arrest, the survival rate until discharge is said to be less than 10% [6]. 

This report describes a case of hypoxic encephalopathy due to resuscitation after 19 minutes of cardiopulmonary arrest due to ventricular fibrillation. In addition, the neurological prognosis of this case was poor according to the prediction of the European Resuscitation Council and the European Intensive Care Society Guidelines 2021 [7]. However, the patient recovered to the extent that he could undergo rehabilitation for activities of daily living (ADL) and return to work at a convalescent hospital, aiming to become independent in walking while improving the disuse syndrome through short-term, high-frequency interventions and changing the assistive device according to muscle output. This report followed the Case Report (CARE) guidelines and the Strengthening the Reporting of Observational Studies in Epidemiology (STROBE) statement.

This case was presented at the 27th Shizuoka Prefecture Physical Therapy Academic Conference on June 23, 2024.

## Case presentation

A 29-year-old man, who was independent in ADL before the onset of the disease, suffered cardiopulmonary arrest while in a sauna, and cardiopulmonary resuscitation was started about 13 minutes later. He was a pharmacist and his past history included Wolff-Parkinson-White (WPW) syndrome which had been followed up until he graduated from elementary school. 

Analysis of the automated external defibrillator showed that it was ventricular fibrillation, and one defibrillation was performed after six minutes, and the heartbeat resumed. He was then brought to our hospital by ambulance, and an electrocardiogram was used to diagnose him as having WPW syndrome type B. The blood data at the time of arrival showed a potential of hydrogen (pH) of 7.251, carbon dioxide partial pressure (pCO2) of 38.2 mmHg, oxygen partial pressure (pO2) of 62.9 mmHg, bicarbonate ion (HCO3) of 16.8 mmol/L, and base excess (BE) of -9.7 mmol/L, indicating hypoxemia and metabolic acidosis. In addition, the patient also had high levels of cardiac markers, including brain natriuretic peptide (BNP) 20.1 pg/mL, creatine kinase (CK) 690 U/L, troponin T 0.462 ng/mL, and CK-MB mass 13 ng/mL (Table 1). Ejection fraction was 35%, and myocardial wall motion was hypokinesis in all directions on echocardiography. The patient was placed on mechanical ventilation and temperature management (36°C for 72 hours) the same day.

**Table 1:** Blood sampling data at time of admission pCO2: carbon dioxide partial pressure; pO2: oxygen partial pressure; HCO3: bicarbonate ion

Test	Result	Reference range
White Blood Cell	138	33-86（10^2^/μL）
Red Blood Cell	478	435-555（10^4^/μL）
Hemoglobin	14.8	13.7-16.8（g/dL）
Potassium	3.3	3.6-4.8（mmol/L）
Sodium	138	138-145（mmol/L）
Brain Natriuretic Peptide (BNP)	20.1	<18.4（pg/mL）
Troponin T	0.462	<0.014（ng/mL）
Creatine Kinase	690	59-248（U/L）
Creatine Kinase-MB Mass	13	<5（ng/mL）
Albumin	4.3	4.1-5.1（g/dL）
Serum creatinine	1.41	0.65-1.07（mg/dL）
D-dimer	4.6	<1（μg/mL）
pH	7.251	7.35-7.45
pCO2	38.2	35-45（mmHg）
pO2	62.9	80-100（mmHg）
HCO3	16.8	22-26（mmol/L）
Base Excess	-9.7	-2-2（mmol/L）

**Figure 1:**
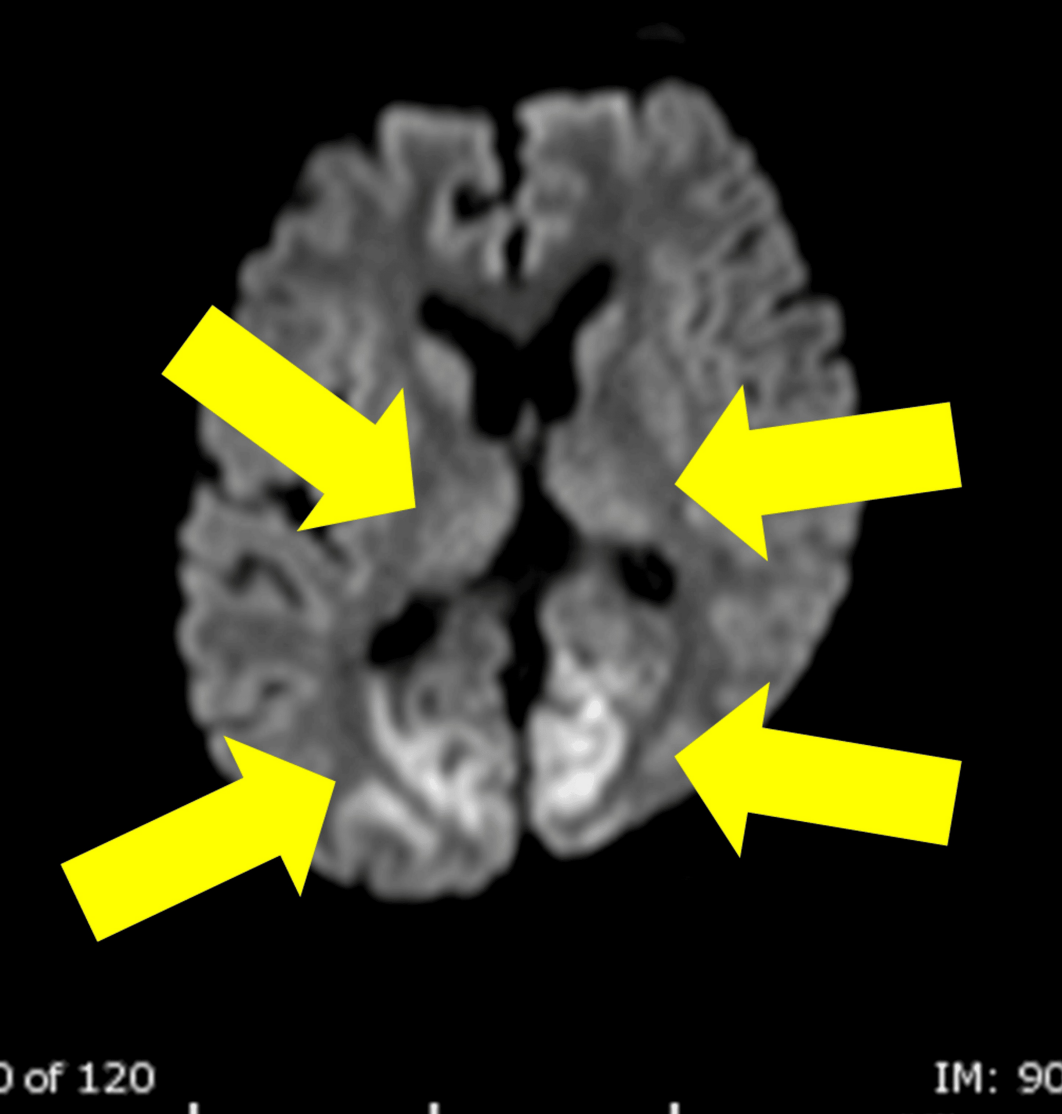
MRI image of the head; DWI high signals were observed in the bilateral occipital and medial thalamus DWI: diffusion-weighted imaging

On Hospital Day (HD) 2, generalized convulsions occurred, and the patient was diagnosed with status epilepticus based on the results of the electroencephalogram. On head MRI, diffusion-weighted imaging (DWI) revealed high signals in the bilateral occipital and frontal lobes and medial thalamus (Figure 1). In addition, neuron-specific enolase (NSE) was 101 ng/mL. Based on the high NSE level, the epileptic seizure, and the head MRI findings, the neurologist diagnosed the patient as having a modified Rankin Scale (mRS) of 5 and a Cerebral Performance Category (CPC) of 4. As there was no prospect of improvement in his level of consciousness, a tracheotomy was performed on HD 18. However, the patient could be weaned from the ventilator on HD 52. As he had frequent status epilepticus in the early stages of hospitalization, he was repeatedly given medication adjustments, and from HD 30 onwards, he was switched from intravenous anesthetics to oral medication, and his level of consciousness gradually improved.

After weaning from the ventilator, we changed to a speech cannula on HD 74, implanted an implantable cardioverter-defibrillator on HD 79, removed the tracheostomy cannula on HD 110, and transferred the patient to a rehabilitation hospital on HD 123.

Rehabilitation intervention

Physiotherapy was started on HD 3 to promote early mobilization but was terminated on HD 15 due to poor consciousness level. Subsequently, physiotherapy was started again on HD 27 due to improved consciousness levels. At the time of the initial intervention (on HD 27), the patient's vital signs were as follows: blood pressure 126/83 mmHg, pulse rate 89 beats/minute, oxygen saturation 98% (biphasic positive airway pressure mode, inhaled oxygen concentration 30%, end-expiratory positive pressure 5 cmH2O, pressure support 10 cmH2O, respiratory rate 10 breaths/minutes), tidal volume 531 ml, and respiratory rate 10 breaths/minute. Intermittent rales were heard in the right middle lobe and left upper lobe. Although the patient was in a state of Glasgow Coma Scale (GCS) E4/VT/M6, there were fluctuations in the level of consciousness, sometimes slipping to E2-3 during the intervention. The Brunnstrom Recovery Stage (BRS) was right III-I-III/left III-I-III, the gross lower limb muscle strength was Manual Muscle Testing (MMT) 1, and the Barthel Index (BI) was 0 points.

The period from the first physiotherapy intervention to weaning from the ventilator was targeted at getting the patient to be able to ride in a wheelchair. At the beginning of the intervention, the patient's physical strength had declined due to the effects of long-term bed rest, and the limit was sitting at the top of the bed for five minutes. In addition, the patient's condition was unstable, with a decline in consciousness level and the appearance of convulsive seizures. Therefore, we performed the range of motion (ROM), muscle strengthening, and edge-sitting exercises for 20 minutes twice daily. The edge-sitting exercises were carried out for 5-15 minutes while listening to the patient's feelings of fatigue. The respiratory rate during the sitting position was 20-25 times per minute, the tidal volume was 280-350 ml, and the blood pressure was in the 120-130 mmHg range, with no significant increase or decrease compared to the supine position. In addition, after consulting with the occupational therapist, occupational therapy was also performed, including joint ROM exercises, upper limb muscle strengthening exercises, and sitting position exercises for 20 minutes twice a day.

From the time he was weaned from the ventilator (HD 52) until he was transferred to another hospital (HD 123), the focus of his physiotherapy was on getting him out of bed. On HD 58, he started standing and using a wheelchair. After that, he moved on to walking practice, but because his trunk and lower limbs had muscle strength of MMT 1, he started walking with a body weight support walker on HD 67 to improve his standing posture and unload his lower limbs. In the early stages, he practiced walking while being unloaded by 40-50 kg. However, as his bilateral quadriceps muscles improved to MMT 2, he began practicing bilateral long-leg walking from HD 76. From HD 93, he was able to walk 50 m with assistance from behind. 

**Figure 2:**
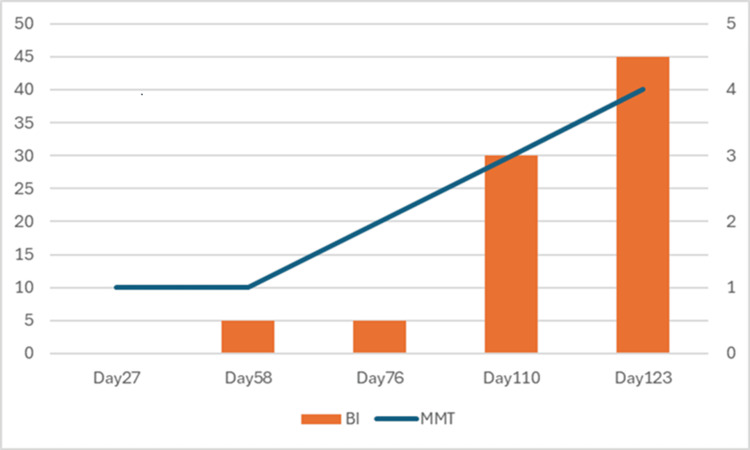
Changes in muscle strength and ADL ADL: activities of daily living; BI: Barthel Index; MMT: manual muscle testing

As a result of continuing to practice walking, the MMT of both quadriceps femoris improved to 3, so walking with elastic bandages on both knee joints was started on HD 110, and from HD 118, walking with light assistance was possible. At the time of transfer to a rehabilitation hospital (HD 123), the BRS was IV-III-IV on both sides, MMT3-4 for both lower limbs and walking was possible for 50 m with light assistance from behind the armpit without any walking aids, and he was able to walk 10 meters with supervision. mRS was 4, and CPC was 3. The BI improved to 45 points (Tables 2, 3) (Figure 2). 

**Table 2:** Changes in physical therapy assessment

Scales	First result	Final result
Glasgow Coma Scale	E4/VT/M6	E4/V5/M6
Brunnstrom Recovery stage	Ⅲ/Ⅰ/Ⅲ	Ⅳ/Ⅲ/Ⅳ
Manual Muscle Testing	iliopsoas muscle1 Quadriceps muscle 1 Tibialis anterior muscle 1 Triceps surae 1	iliopsoas muscle 4 Quadriceps muscle 4 Tibialis anterior muscle 4 Triceps surae 3
Barthel Index	0	45 （Feeding 10/Transfer 5/Mobility on level surfaces 10/ Bowel control 10/Bladder control 10）
modified Rankin Scale	5	4
Cerebral Performance Category	4	3

**Table 3:** Blood sampling data at time of hospital transfer (Hospital day 123)

Test	Result	Reference range
White Blood Cell	93	33-86（10^2/μL）
Red Blood Cell	420	435-555（10^4/μL）
Hemoglobin	12.9	13.7-16.8（g/dL）
Potassium	3.8	3.6-4.8（mmol/L）
Sodium	142	138-145（mmol/L）
Brain Natriuretic Peptide (BNP)	4.4	<18.4（pg/mL）
Creatine Kinase	35	59-248（U/L）
Creatine Kinase-MB Mass	3	<5（ng/mL）
Serum creatinine	0.78	0.65-1.07（mg/dL）
D-dimer	1.2	<1（μg/mL）

In the higher brain function tests, the Mini-Mental State Examination score (MMSE) was 24 points, the Hasegawa Dementia Scale -Revised score was 24/30 points, the Rivermead Behavioral Memory Test score was 5/24 points for the standard profile and 2/12 points for the screening profile, and memory impairment was observed. In occupational therapy, interventions were mainly focused on ADL practice such as turning over in bed, getting up, and practicing eating. Furthermore, the ward nurses were asked to increase the number of times the patient was in a wheelchair in the ward and to extend the time spent in the wheelchair in order to improve the patient's ADL. On HD 123, he was transferred to a rehabilitation hospital for the recovery period and is currently continuing outpatient rehabilitation to return to work, and his mRS has improved to 3.

## Discussion

This was a case in which the patient suffered from hypoxic encephalopathy due to cardiopulmonary arrest for 19 minutes caused by ventricular fibrillation, and both the patient's life prognosis and neurological prognosis were expected to be poor. The results of the mRS and CPC indicated that the patient was expected to have impaired function. However, the presence or absence of multiple organ failure affects the life prognosis [8], and age also affects functional prognosis [9]. In this case, both life and functional prognoses gradually improved.

From the first rehabilitation intervention to weaning from the ventilator, short-term, high-frequency interventions were carried out for 20 minutes per session, two to four times a day, while monitoring blood pressure, pulse rate, and tidal volume. The Guidelines for Rehabilitating Critically Ill Patients also recommend multiple rehabilitation interventions [10]. In addition, the more hours of rehabilitation intervention there are in a day, the more improvements can be made in muscle strength [11], walking ability, and ADL [12]. Furthermore, even low-load stimulation can increase maximum muscle strength [13], so low-load but high-frequency intervention can improve posture-holding ability and lower-limb muscle strength, enabling the patient to sit up for at least 15 minutes.

From the time of weaning from the ventilator until transfer to another hospital, the patient actively got out of bed, changing the walking aid according to the amount of walking assistance required. In the early stages of walking, we used an unweighting walker. We adjusted the weight-bearing load while practicing walking, which helped to create a sense of weight-bearing and a trigger for the central pattern generator drive necessary for walking [14], and we were able to improve the walking rhythm and muscle contraction. After that, since the muscle output of the quadriceps femoris improved and the standing posture improved, we moved on to two-motion gait training with bilateral long-legged orthoses and assisted backward walking. Two-motion gait training with long-legged orthoses can promote more muscular contraction of the lower limb muscles [15,16], leading to improved muscle strength and improved ADL at the time of transfer to our hospital. As there is a positive correlation between the walking items and the Functional Independence Measure for stairs at the time of transfer to a convalescent hospital and the use of long-legged orthoses during the acute phase of treatment [17], long-legged orthoses are important during the acute phase of treatment. The creation of a long lower limb orthosis for the patient and gait training during hospitalization in an acute care hospital can affect the patient's ability to walk and climb stairs and the rate of orthosis removal when they are discharged from a rehabilitation hospital in the recovery period [18], so it is also important to create an environment where orthoses can be made from the acute care stage. In this case, the physiotherapy focused on getting out of bed, and the occupational therapy focused on practicing ADL. Furthermore, by working together with the ward nurses to extend the time spent in a wheelchair and by working with a range of different professionals, we were able to improve the patient's ADL.

Since prolonged seizures indicate a poor neurological prognosis [19], it was also important that we were able to prevent secondary brain damage by reducing the frequency of status epilepticus through temperature control therapy and medication adjustment [20].

## Conclusions

In this case, the patient had a 19-minute cardiopulmonary arrest, extensive findings of hypoxic encephalopathy, and high NSE levels, and both the patient's life and neurological prognosis were poor. In addition, his muscle strength and physical strength had deteriorated significantly due to long-term intensive care unit admission and ventilator management. In addition to treatment and medication adjustments by doctors, short-term, high-frequency interventions by physical therapy were carried out to improve muscle strength and physical strength and to promote weaning. As a result of promoting walking practice while selecting assistive devices that match muscle output while collaborating with multiple professions, improvements in ADL were achieved at the time of transfer to the rehabilitation hospital.
